# Molecular Detection of Pathogenic *Leptospira* sp. in Cetaceans from the Brazilian Coast

**DOI:** 10.1155/2023/7041089

**Published:** 2023-06-23

**Authors:** Felipe D'Azeredo Torres, Ana Luiza dos Santos Baptista Borges, Pedro Volkmer de Castilho, Cristiane Kolesnikovas, Camila Domit, Jonathas dos Santos, Waltyane Alves Gomes Bonfim, Vitor Luz Carvalho, Carla Beatriz Barbosa, Vanessa Lanes Ribeiro, Filipe Anibal Carvalho-Costa, Maria Isabel Nogueira Di Azevedo, Walter Lilenbaum

**Affiliations:** ^1^Laboratory of Veterinary Bacteriology, Biomedical Institute, Fluminense Federal University, Niterói, Rio de Janeiro, Brazil; ^2^Laboratory of Zoology, University of the State of Santa Catarina, Laguna, Santa Catarina, Brazil; ^3^R3 Animal Association, Florianópolis 88061-500, Santa Catarina, Brazil; ^4^Laboratory of Ecology and Conservation, Universidade Federal do Paraná, Paraná, Brazil; ^5^Beach Monitoring Project of Sergipe-Alagoas Basin, Vichi Management Projects, Aracaju, Sergipe, Brazil; ^6^Biota Conservation Institute, Maceió, Alagoas, Brazil; ^7^Association for Research and Preservation of Aquatic Ecosystems, Caucaia, Brazil; ^8^Argonauta Institute for Coastal and Marine Conservation, Ubatuba, São Paulo, Brazil; ^9^Biopesca Institute, Praia Grande, São Paulo, Brazil; ^10^Laboratory of Epidemiology and Molecular Systematics, Oswaldo Cruz Institute, Rio de Janeiro, Brazil

## Abstract

Leptospirosis is a zoonosis with ubiquitous distribution caused by spirochetes belonging to the genus *Leptospira* sp., endemic mainly in tropical and subtropical regions of the world and capable of infecting domestic animals, free-living animals, and humans. Although well documented in terrestrial animals and humans, little information is available on its distribution and impact on marine animals. There are few studies assessing cetaceans' health status, and even scarcer are those focused on leptospirosis research. In this context, considering the One Health approach, the present study aimed to investigate the occurrence of pathogenic *Leptospira* sp. in cetaceans on the Brazilian coast. Kidneys of 142 cetaceans belonging to 19 species were collected. DNA was extracted, and the diagnosis was performed by *LipL*32-polymerase chain reaction. Genetic characterization was conducted based on *secY* gene sequencing. Pathogenic *Leptospira* sp. DNA was detected in 14.8% (21/142) of the tested cetaceans, with coastal species presenting a significantly higher frequency (*p-*value = 0.03) of infected individuals (25%, 17/68) than oceanic species (7.5%, 4/53). It was possible to amplify and sequence three strains (one for *Sotalia guianensis*, one for *Stenella clymene*, and one for *Pontoporia blainvillei*), all of them identified as *Leptospira interrogans*, with high similarity with sequences from Icterohaemorrhagiae serogroup. Phylogenetic analysis revealed sequences from the present study grouped in species-specific unique clusters but very close to pinnipeds in the same area, evidencing the presence of two distinct haplotypes circulating on marine mammals in the region. We could demonstrate that cetaceans can act as carriers of pathogenic leptospires. Moreover, the proximity with anthropogenic areas could play an important role in leptospirosis' dynamics of transmission in a One Health context.

## 1. Introduction

Marine mammals live long, grow slowly, and have low fecundity. These features make them good sentinels' species and indicators of ocean health because they are directly exposed to environmental changes caused by natural and anthropogenic causes [1, 2]. Based on the type of stressors influence, like diseases and pathogens found in these free-ranging animals, it is possible to indicate risks to human health, mainly due to the common use of the marine habitat [3, 4]. This information becomes necessary to prevent human threats, especially at the interface between humans and free-living animals [5], fundamental to a One Health context.

Emerging and resurgent diseases, including zoonoses and diseases originating in domestic animals, have had significant impacts on various populations of marine mammals worldwide [6]. These act not only as victims of these zoonoses but also as carriers, moving microorganisms to different geographic locations in the ocean [7]. Among the zoonoses, leptospirosis deserves to be highlighted. It is a systemic bacterial disease caused by pathogenic strains of the genus *Leptospira* sp., endemic in tropical and subtropical regions of the world and capable of infecting domestic and free-living animals, and humans [8, 9].

Although well documented and characterized in terrestrial animals and humans, little information is available on its distribution and impact on marine animals [10] and the significance of the infections for their health or their potential role as intermediate or reservoir hosts of leptospires infecting terrestrial mammals, including humans, still unclear [11]. On the other hand, it has been suggested that all free-ranging animals could be incidental *Leptospira* hosts, including marine mammals [12]. Recently, our group recognized pinnipeds as carriers of pathogenic leptospires on the South and Southeast coast of Brazil, highlighting a possible role in leptospirosis dynamics in the region [13].

Cetaceans were first investigated as possible hosts for *Leptospira* in the 1980s when the authors used the microagglutination test (MAT) on Greenland whales, but they were all nonreactive [14]. With the advent of molecular techniques, large-scale studies with stranded animals began to deeper investigate *Leptospira* sp. in cetaceans [7] but without a conclusive diagnosis. The first positive results confirming *Leptospira* infection in cetaceans were obtained by kidney culture, first in Argentina [15] and later in Italy [16]. These results reinforce the need to expand studies of leptospirosis in cetaceans in other regions of the world. Different cetacean stranding events were monitored along the coast of the Philippines, and cetaceans of 14 species presented a high positivity rate (64%, 18/28) [5].

Despite its large coastal region of approximately 8,000 km [17], there are few studies in Brazil regarding cetaceans' health status, and even scarcer are those focused on leptospirosis research, with no confirmation of the infection [18, 19]. As an important zoonosis, more studies regarding those animals are essential for a better understanding of their role in the environment–animal–human interface [20]. In this context and considering the One Health approach and the Ocean Decade challenge (UNESCO/2017), the aim of the present study was to investigate the occurrence of pathogenic *Leptospira* sp. in cetaceans on the Brazilian coast.

## 2. Materials and Methods

### 2.1. Ethical Approval and Collection of Samples

To carry out this project, authorizations were obtained from the Ethics committee of Fluminense Federal University number 1058220720 (granted on October 9, 2020) and from the Biodiversity Authorization and Information System (SISBIO) number 74847-1 provided by Chico Mendes Institute for Biodiversity Conservation. Kidney's samples of 142 dead cetaceans, belonging to 19 species, were collected by institutions participating in the stranding network of marine animals on the South to Northeast coast of Brazil from Santa Catarina to Ceará states between the years 2015 and 2022 (*Supplementary 1*). Cetaceans were rescued daily through monitoring actions directly and opportunistically through notifications by the local community.

### 2.2. Individual Data of Collected Animals

All individuals registered by the network were characterized according to (1) species, (2) sex (based on microscopic evaluation of the gonads), (3) age group, (4) decomposition code^*∗*^, (5) grounding site by Eco-Region (Southwestern Atlantic X Warm Temperate Southwestern Atlantic V) according to Spalding et al. [21], and (6) Habitats (coastal X oceanic).

Data regarding the histopathology of renal tissues were provided by the partner institutions and used for the classification of the animals into the status: (1) Kidney A = individuals without alterations compatible with renal pathology associated with leptospirosis, and (2) Kidney B = individuals with alterations compatible with renal pathology associated with leptospirosis (according to Torres et al. [13]). The following inclusion criteria were adopted: animals of any sex, any age group, ecoregion, preferential habitats, and kidney status. Decomposition codes were just accepted between 2 and 4 [22].

### 2.3. DNA Extraction and Molecular Diagnosis

Molecular analyzes were performed as previously described by D'Azeredo Torres et al. [13]. Duplicates of kidney samples were obtained at the time of necropsy and stored in sterile 2.0 mL microtubes at −20°C and reserved for molecular analysis. DNA extraction was performed using the Dneasy® Blood & Tissue Kit (Qiagen, California, USA), according to the manufacturer's instructions. Specific primers of the *Lip*L32 gene, reported to be present in pathogenic leptospires [23], were used for the reactions. Reactions were performed as previously described by Hamond et al. [24]. For each test, ultrapure water was used as a negative control in all reactions, while 10 fg of DNA extracted from *Leptospira interrogans* serovar Copenhageni (Fiocruz L1-130) was used as a positive control. The polymerase chain reaction (PCR) products were analyzed by electrophoresis in 1.5%–2% agarose gel after gel red staining and then visualized under UV light.

### 2.4. Sequencing and Phylogenetic Analysis

Samples positive for *Lip*L32-PCR were submitted to a nested PCR targeting a fragment of the *sec*Y gene (410 bp). An initial reaction was conducted using primers secY_outerF (5′-ATGCCGATCATTTTTGCTTC-3′) and secY_outerR (5′-CCGTCCCTTAATTTTAGACTTCTTC-3′), according to the conditions described by Ahmed et al. [25]. Posteriorly, amplicons were included in a second reaction using primers secY_inner_F (5′-CCTCAGACGATTATTCAATGGTTATC-3′) and secY_inner_R (5′-AGAAGAGAAGTTCCACCGAATG-3′) [26].

The *sec*Y amplicons were purified with the Wizard® SV Gel Kit and PCR Clean-Up System (Promega, USA), according to the manufacturer's instructions and intended for sequencing. Sequencing reactions were performed using the Big Dye Terminator v. 3.1 Cycle Sequencing Kit (Applied Biosystems, USA) on a 3100 automatic DNA sequencer according to the manufacturer's instructions. Regarding sequences analysis, Pairwise/Blast/NCBI software, SeqMan v. 7.0, ClustalW v. 1.35 (12), and BioEdit v. 7.0.1 [27] were used to edit and analyze the sequences. A maximum likelihood (ML) tree was constructed using the Tamura–Nei model (TN92) in MEGA X software [28], as it was determined to be the best-fitting model of DNA substitution using the Bayesian information criterion. Genetic distances were calculated using the TN92 model on MEGA X.

### 2.5. Statistical Analysis

The results obtained for eco-regions, preferential habitats, and clinical status were analyzed by Qui-Square Test in Microsoft Software (Excel), whereas age, sex, and decomposition code were analyzed in general linear model (GLM) in R Studio Software. Because it is an opportunity sampling, there were variations between the number of individuals sampled of each species, and therefore this parameter was not statistically evaluated to avoid research bias. All participating institutions used the same sampling protocol, and for the correct taxonomic identification and habitat classification, one specialized professional critically reviewed all individuals inserted in this survey [29, 30].

## 3. Results

### 3.1. Molecular Detection of Pathogenic *Leptospira* spp. and Kidney Status

Pathogenic *Leptospira* sp. DNA was detected in 14.8% (21/142) of the tested cetaceans, with a higher proportion in *Sotalia guianensis* (47.6%, 10/21) and *Pontoporia blainvillei* (33.4%, 7/21) (Table 1). Coastal species presented a higher frequency of infected individuals (25%, 17/68) than oceanic species (7.5%, 4/53) (*p-*value = 0.03). Otherwise, the ecoregions tropical southwestern Atlantic and warm temperate southwestern Atlantic presented almost the same frequency, 16% (11/58) and 14% (10/63), respectively, with no significant difference (*p-*value > 0.05).

Leptospiral DNA detection varied according to decomposition categories accepted in this study, with COD 2%–17.6% (12/68), COD 3%–12.5% (9/72), and no detection observed in COD 4 (0/2). In terms of sex, no significant differences were observed (males 14.3% and females 16.9%). Regarding age group, calves presented a higher prevalence, 23.3% (7/30), when compared with juveniles 13.7% (7/51) and adults 13.2% (7/53). Sex and age group were not correctly defined in six cases, given an indeterminate result for each one. Despite these subtle differences, all comparisons between categories were not significant in GLM.

Kidney histopathology was analyzed in 55.6% (79/142) of animals, and of them, 86.1% (68/79) did not present anatomopathological relevant findings, so they were classified as Kidney A, with the others 13.9% (11/79) as Kidney B. Among the PCR-positive kidneys (*n* = 21), 11 (52.4%) did not present histopathological lesions, while four (19%) of PCR-positive kidneys showed histopathological lesions. Histopathological findings were represented by the presence of multifocal areas of moderate hemorrhage and congestion (3/4) and mild multifocal renal tubular necrosis (1/4). In the remaining positive individuals, 28.6% (6/21), it was not possible to perform histopathology due to the tissue's autolysis. No significant differences were seen in these categories. Detailed information for each animal included in this study is shown in *Supplementary 1*.

### 3.2. Sequencing and Phylogenetic Analysis

*Lip*L32-PCR positive samples (*n* = 21; *S. guianensis: n* = 10, *P. blainvillei: n* = 7, *Tursiops truncatus: n* = 2, *Stenella clymen*e: *n* = 1 and *Steno bredanensis: n* = 1) were submitted to *sec*Y nested-PCR. From those, it was possible to amplify and sequence three amplicons (424 bp) from *S. guianensis, P. blainvillei*, and *S. bredanensis*. Sequences were deposited on GenBank under accession numbers OP978284–OP978286. After Pairwise/Blast/NCBI comparisons with GenBank *sec*Y gene dataset, all three sequences were identified as *L. interrogans* with an identity >99%.

**Figure 1:**
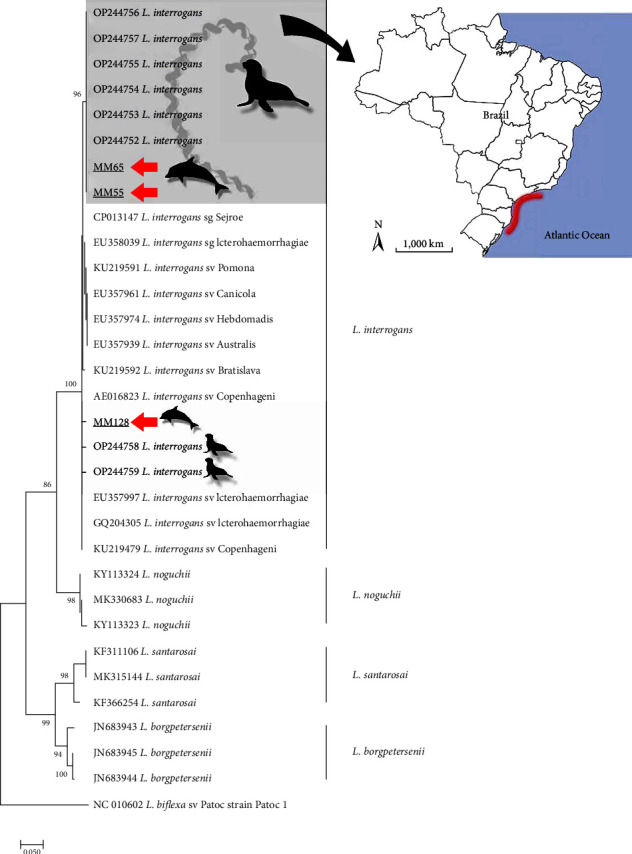
Maximum likelihood phylogenetic tree inferred from partial *sec*Y gene sequences from the present study (red arrow) and GenBank sequences from the main pathogenic *Leptospira* species. Sequences from marine mammals are represented by vectors. The cluster formed by a unique *Leptospira interrogans* haplotype from pinnipeds and cetaceans from the southeast/south littoral of Brazil (map) is highlighted in gray. *Leptospira biflexa* serovar Patoc is the outgroup taxa.

In order to confirm species identity and evaluate genetic correlations with other strains, phylogenetic analysis based on ML-TN92 was conducted with sequences from the present study (*n* = 3) and GenBank sequences from the main pathogenic *Leptospira* species. The three sequences from the present study (MM55, MM65, and MM128) clustered in a highly supported clade (bootstrap = 100%) with *L. interrogans* sequences, clearly separated from the other pathogenic species (Figure 1). Interestingly, but not surprisingly, two of the sequences were 100% similar to other sequences (OP244752–OP244756) of *L. interrogans* from pinnipeds from the same geographical region (Figure 1). Moreover, the sequence MM128 was 100% similar to two other *L. interrogans* sequences from pinnipeds stranded on the Brazilian coast (OP244758 and OP244758), evidencing the presence of two distinct haplotypes circulating on marine mammals in the southern Atlantic Ocean. Regarding serological classification, sequences from the present study showed high similarity (>99%) with a great variety of serovars, including Icterohaemorrhagiae, the most associated with human acute disease (Figure 1).

## 4. Discussion

The study of *Leptospira* infection in cetaceans is still challenging, and the results obtained in the present work improve the current knowledge and highlight important health concerns in the marine environment. It is important to emphasize that the observed occurrence rate (14.8%) is unprecedented in the biodiversity of the Atlantic Ocean. Moreover, it is the first time *Leptospira* infection has been reported in cetaceans on the Brazilian coast. Additionally, despite being genetically very close to known strains, sequences from the present study clustered together with *Leptospira* spp. sequences from pinnipeds from the same geographical localization [13], evidencing distinct but exclusive *L. interrogans* haplotypes of marine mammals in the oceanic biome in the region. Since many of these hosts did not present marked kidney lesions on histopathology, a possible host–parasite adaptation can be suggested, as previously demonstrated in other hosts, such as livestock [31, 32].

As far as we know, all cetaceans ever tested by MAT presented negative [14, 19]. This lack of seroreactivity may be explained by the fact that the test could present low sensitivity for those animals [6]. Another important concept is a possible adaptation of strains to the host that may not stimulate an efficient humoral response, making MAT inefficient [33].

The first report of *Leptospira* infection in cetaceans was obtained by a positive culture of a kidney sample from a stranded southern right whale (*Eubalaena australis*) on the coast of Argentina. In addition to this unprecedented data, the ability of leptospires to grow in salty waters was also reported, modifying the understanding of the epidemiology of this zoonosis [15]. In a similar situation, on the coast of Italy, another positive culture was obtained from a common bottlenose dolphin (*T. truncatus*) [11]. In common, the two studies obtained one single positive sample from carcasses stranded on the coast with a moderate/advanced stage of decomposition. Culturing of leptospires is the most specific method to demonstrate their presence [9] and is considered the gold standard for the diagnosis of the infection. However, the bacteria are fastidious, and the method is laborious and longstanding, so it is still a challenge to obtain pure cultures in field studies [34].

From the 19 species of cetaceans from different geographic locations of the Brazilian coast (south, southeast, and northeast regions), positive results were obtained in five species, of which three (*S. guianensis*, *P. blainvillei*, and *S. clymene*) constitute the first record of *Leptospira* infection. The variety of sampled species and the remarkable results were just possible since we were able to test frozen tissues. Molecular tools are better to detect infected animals when compared to serology, culture, or immunohistochemistry [5, 35]. Besides, PCR helps in research in which individuals normally strand dead, and often in moderate/advanced decomposition code. Moreover, in some hosts, *Leptospira* infection may not lead to an important serological response, so they should be diagnosed by direct demonstration of the agent or its DNA [36]. Apparently, it may also be the case for cetaceans.

In the absence of clinical signs records, we made an attempt to access information about clinical status by kidney histopathology. Unfortunately, we could perform it in only half (55.6%) of the carcasses, so the results could only offer a limited view of kidney damage. Lesions were only seen in four infected animals and were characterized by hemorrhage, congestion, and, in one case, necrosis. Leptospirosis in marine mammals is referred to lead to interstitial nephritis, as seen in pinnipeds, mustelids, and cetaceans [5, 37–39]. In this way, due to the low number of lesions observed, it is not possible to reliably affirm that the observed lesions were indeed determined by leptospirosis.

Noteworthy observe that coast species are more probable to be infected than oceanic species (*p-*value = 0.03), and the coastal species *S. guianensis* and *P. blainvillei* presented as infected in this study. It should be noted that some species, such as *T. truncatus* and *S. bredanensis*, have both coastal and oceanic behaviors along the Brazilian coast [40, 41]; therefore, despite the international classification as oceanic [42], regional characteristics must be considered, and the results could be even more expressive. The real meaning of this finding is still an object of speculation. In pinnipeds, it was reported that animals who inhabit areas surrounded by more human-populated areas have higher titers against *Leptospira* sp. [43]. It is also believed that contact with urban animals (dogs, cats, and rats) favors the sharing of pathogens, and they can be infected by diseases with zoonotic potential [44]. It is also noteworthy that these bacteria grow in salt water, and it is possible that the source of coastal species infection could be land-based effluents [5]. Remains a challenge to understand their ability to remain viable and infect oceanic species, like those reported in this paper (*T. truncatus*, *S. clymene*, and *S. bredanensis*), and other oceanic species already reported [15, 16].

We reported the presence of pathogenic *Leptospira* in five different species of cetaceans in the South Atlantic Ocean based on molecular diagnosis and genetic characterization of amplicons, providing new data about hosts and geographical localization for this bacterial species. Moreover, it was shown the presence of two distinct haplotypes circulating on marine mammals on the Brazilian coast, both exclusive to these hosts. Importantly, a higher occurrence affecting coastal species was observed, which deserves attention due to the proximity to humans and other terrestrial animals, corroborating, even more, the complexity of the transmission dynamics of pathogenic leptospires in a One Health world.

## Figures and Tables

**Table 1:** Summarized results of cetacean's species sampled in the Brazilian coast (*n* = 142) submitted to molecular analysis as part of pathogenic *Leptospira* sp. investigation.

Eco-region	Hosts per Brazilian region		*Lip*L32-PCR	
	Scientific name	Common name	Negative	Positive
Warm Temperate Southwestern Atlantic (WAR)	Northeast			
	*Balaenoptera physalus*	Fin whale	1	
	*Feresa attenuata*	Pygmy killer whale	5	
	*Grampus griseus*	Risso's dolphin	1	
	*Globicephala macrorhynchus*	Short-finned pilot whale	4	
	*Kogia breviceps*	Pygmy sperm whale	2	
	*Kogia sima*	Dwarf sperm whale	5	
	*Megaptera novaeangliae*	Humpback whale	4	
	*Peponocephala electra*	Melon-headed whale	4	
	*Stenella attenuata*	Pantropical spotted dolphin	1	
	*Stenella clymene*	Clymene dolphin	1	1
	*Stenella coeruleoalba*	Striped dolphin	2	
	*Sotalia guianensis*	Guiana dolphin	30	9
	*Stenella longirostris*	Spinner dolphin	2	
	*Tursiops truncatus*	Common bottlenose dolphin	1	
	Total		63	10
	Southeast			
Southwestern Atlantic (SAR)	*Balaenoptera autorostrata*	Common minke whale	1	
	*Kogia breviceps*	Pygmy sperm whale	1	
	*Pontoporia blainvillei*	La Plata dolphin	8	3
	*Steno bredanensis*	Ough-toothed dolphin	1	
	*Sotalia guianensis*	Guiana dolphin	10	1
	Total		21	4
	South			
	*Balaenoptera autorostrata*	Common minke whale	1	
	*Eubalaena australis*	Southern right whale	1	
	*Kogia breviceps*	Pygmy sperm whale	1	
	*Kogia sima*	Dwarf sperm whale	1	
	*Megaptera novaeangliae*	Humpback whale	1	
	*Pontoporia blainvillei*	La Plata dolphin	13	4
	*Steno bredanensis*	Ough-toothed dolphin	1	1
	*Stenella coeruleoalba*	Striped dolphin	1	
	*Stenella frontalis*	Atlantic spotted dolphin	3	
	*Sotalia guianensis*	Guiana dolphin	2	
	*Stenella longirostris*	Spinner dolphin	1	
	*Stenella* sp.	Without species classification	1	
	*Tursiops truncatus*	Common bottlenose dolphin	10	2
	Total		37	7
	*Total*		*121*	*21*

PCR, polymerase chain reaction.

## Data Availability

The data used to support the findings of this study are included within the article.

## Supplementary Materials

Supplementary Materials Results of kidney tissues molecular analysis from cetaceans sampled in Brazilian coast.
