# Supplementary material for: Molecular Detection of Pathogenic Leptospira sp. in Cetaceans from the Brazilian Coast
Source: Transbound Emerg Dis. 2023 Jun 23;2023:7041089. doi: 10.1155/2023/7041089 (PMC12016789; doi:10.1155/2023/7041089)
Supplement: Supplementary Materials — Results of kidney tissues molecular analysis from cetaceans sampled in Brazilian coast. [file 7041089.f1.docx]

| **ID** | ***Lip*L32**  **PCR** | ***sec*Y sequencing** | **Status** | **Morphological Kidney description** | **Species** | **Habitat** | **City/State** | **Eco-Region** | **Sex** | **Age** | **COD** |
| --- | --- | --- | --- | --- | --- | --- | --- | --- | --- | --- | --- |
| MM001 | - |  | A | Mild Renal Necrosis | *Tursiops truncatus* | Oceanic | Imbituba/SC | SAR | F | A | 3 |
| MM002 | - |  | A | Moderately and diffusely engorged vessels | *Eubalaena australis* | Oceanic | Imbituba/SC | SAR | M | F | 3 |
| MM007 | - |  | A | Moderate Autolysis | *Tursiops gephyreus* | Coastal | Laguna/SC | SAR | F | J | 3 |
| MM008 | - |  | A | Mild renal necrosis. | *Stenella frontalis* | Coastal | Imbituba/SC | SAR | M | J | 2 |
| MM009 | - |  | N/A | N/A | *Tursiops truncatus* | Oceanic | Laguna/SC | SAR | F | I | 4 |
| MM010 | - |  | A | Intense multifocal hyperemia | *Pontoporia blainvillei* | Coastal | Laguna/SC | SAR | F | A | 3 |
| MM012 | - |  | A | Health tissue | *Tursiops truncatus* | Oceanic | Imbituba/SC | SAR | M | A | 3 |
| MM014 | - |  | A | Intense diffuse hyperemia. | *Stenella coeruleoalba* | Oceanic | Laguna/SC | SAR | F | A | 3 |
| MM015 | - |  | A | Intense multifocal hyperemia. | *Kogia breviceps* | Oceanic | Laguna/SC | SAR | F | A | 2 |
| MM019 | - |  | B | Moderate diffuse hyperemia with moderate focally extensive subcapsular hemorrhage | *Kogia sima* | Oceanic | Garopaba/SC | SAR | M | A | 3 |
| MM020 | - |  | A | Health tissue | *Tursiops truncatus* | Oceanic | Imbituba/SC | SAR | F | J | 3 |
| MM023 | - |  | A | cortical with moderate diffuse hyperemia | *Sotalia guianensis* | Coastal | Biguaçu/SC | SAR | M | J | 3 |
| MM029 | - |  | A | Health tissue | *Steno bredanensis* | Oceanic | Imbituba/SC | SAR | F | J | 3 |
| MM030 | - |  | A | in the interstitium there is discrete lymphoplasmic and eosinophytic infiltrate and multifocal concentrated congestion. | *Pontoporia blainvillei* | Coastal | Laguna/SC | SAR | F | A | 3 |
| MM046 | + | LSQ | A | Health tissue | *Pontoporia blainvillei* | Coastal | Florianópolis/SC | SAR | F | A | 2 |
| MM047 | - |  | N/A | Autolisys | *Tursiops gephyreus* | Coastal | Florianópolis/SC | SAR | F | A | 3 |
| MM048 | - |  | N/A | N/A | *Pontoporia blainvillei* | Coastal | Florianópolis/SC | SAR | M | J | 3 |
| MM049 | - |  | N/A | N/A | *Pontoporia blainvillei* | Coastal | Florianópolis/SC | SAR | F | F | 2 |
| MM050 | + | LSQ | A | Health tissue | *Pontoporia blainvillei* | Coastal | Florianópolis/SC | SAR | M | F | 2 |
| MM051 | - |  | A | Health tissue | *Tursiops truncatus* | Oceanic | Florianópolis/SC | SAR | M | J | 3 |
| MM052 | + | LSQ | A | Health tissue | *Pontoporia blainvillei* | Coastal | São Francisco do Sul/SC | SAR | M | F | 2 |
| MM053 | + | LSQ | A | Rim: median multifocal passive congestion. | *Tursiops truncatus* | Oceanic | Florianópolis/SC | SAR | M | J | 3 |
| MM054 | - |  | A | Health tissue | *Stenella frontalis* | Coastal | Florianópolis/SC | SAR | M | A | 2 |
| MM055 | + | *L. interrogans* | A | Health tissue | *Pontoporia blainvillei* | Coastal | Florianópolis/SC | SAR | M | F | 3 |
| MM056 | - |  | A | discreet congestion | *Pontoporia blainvillei* | Coastal | Florianópolis/SC | SAR | F | J | 3 |

| **ID** | ***Lip*L32**  **PCR** | ***sec*Y sequencing** | **Status** | **Morphological Kidney description** | **Species** | **Habitat** | **City/State** | **Eco-Region** | **Sex** | **Age** | **COD** |
| --- | --- | --- | --- | --- | --- | --- | --- | --- | --- | --- | --- |
| MM057 | + | LSQ | A | Health tissue | *Tursiops truncatus* | Oceanic | Florianópolis/SC | SAR | F | F | 2 |
| MM058 | - |  | A | Mildcongestion | *Pontoporia blainvillei* | Coastal | Florianópolis/SC | SAR | F | J | 3 |
| MM059 | - |  | A | Health tissue | *Pontoporia blainvillei* | Coastal | Florianópolis/SC | SAR | M | F | 3 |
| MM061 | - |  | N/A | N/A | *Tursiops truncatus* | Oceanic | Guaraqueçaba/PR | SAR | F | J | 3 |
| MM062 | - |  | A | Calcification of some medullary tubules, associated with tubular ectasia. | *Tursiops truncatus* | Oceanic | Matinhos/Pontal do sul PR | SAR | F | A | 2 |
| MM063 | - |  | A | moderate diffuse passive congestion. | *Tursiops truncatus* | Oceanic | Matinhos/Pontal do sul PR | SAR | F | J | 2 |
| MM065 | + | *L. interrogans* | B | Mild multifocal renal tubular necrosis with intraluminal hyaline casts and moderate multifocal cysts | *Steno bredanensis* | Oceanic | Paranagua PR | SAR | M | A | 2 |
| MM067 | - |  | N/A | N/A | *Stenella frontalis* | Coastal | Matinhos/Pontal do sul PR | SAR | F | A | 3 |
| MM068 | - |  | A | Moderate multifocal spinal cord region congestion | *Balaenoptera acutorostrata* | Oceanic | Paranagua PR | SAR | F | J | 2 |
| MM069 | - |  | N/A | Autolisys | *Megaptera novaeangliae* | Oceanic | Matinhos/Pontal do sul PR | SAR | F | J | 3 |
| MM070 | - |  | N/A | Autolisys | *Stenella sp.* | Oceanic | Guaratuba PR | SAR | F | J | 3 |
| MM074 | - |  | N/A | N/A | *Pontoporia blainvillei* | Coastal | Guaraqueçaba PR | SAR | I | I | 3 |
| MM075 | - |  | A | Moderate multifocal passive congestion in interstitium | *Stenella longirostris* | Oceanic | Guaratuba PR | SAR | F | I | 3 |
| MM076 | - |  | N/A | Autolisys | *Pontoporia blainvillei* | Coastal | Matinhos/Pontal do sul PR | SAR | F | A | 3 |
| MM077 | - |  | A | Intense multifocal hyperemia. | *Pontoporia blainvillei* | Coastal | Guaratuba PR | SAR | M | I | 3 |
| MM078 | - |  | A | Moderate, diffuse congestion | *Pontoporia blainvillei* | Coastal | Paranagua PR | SAR | M | J | 3 |
| MM085 | - |  | A | Moderate, diffuse congestion | *Pontoporia blainvillei* | Coastal | Guaratuba PR | SAR | F | F | 2 |
| MM086 | - |  | A | Mild to moderate, multifocal congestion is observed. | *Sotalia guianensis* | Coastal | Matinhos/Pontal do sul PR | SAR | M | J | 3 |
| MM087 | - |  | A | Multifocal moderate to severe congestion | *Pontoporia blainvillei* | Coastal | Paranagua PR | SAR | F | J | 2 |
| MM091 | - |  | A | Moderately and diffusely engorged vessels | *Pontoporia blainvillei* | Coastal | Ubatuba/SP | SAR | F | J | 3 |
| MM092 | - |  | A | Mild renal necrosis | *Steno bredanensis* | Oceanic | Ubatuba/SP | SAR | M | A | 2 |
| MM093 | - |  | N/A | Autolisys | *Sotalia guianensis* | Coastal | Ubatuba/SP | SAR | F | J | 3 |
| MM094 | - |  | N/A | Autolisys | *Pontoporia blainvillei* | Coastal | Ubatuba/SP | SAR | M | F | 3 |
| MM095 | - |  | N/A | Autolisys | *Sotalia guianensis* | Coastal | Ubatuba/SP | SAR | F | J | 3 |
| MM096 | + | LSQ | N/A | Autolisys | *Pontoporia blainvillei* | Coastal | Ubatuba/SP | SAR | I | A | 3 |

| **ID** | ***Lip*L32**  **PCR** | ***sec*Y sequencing** | **Status** | **Morphological Kidney description** | **Species** | **Habitat** | **City/State** | **Eco-Region** | **Sex** | **Age** | **COD** |
| --- | --- | --- | --- | --- | --- | --- | --- | --- | --- | --- | --- |
| MM097 | - |  | A | Moderate diffuse hyperemia | *Sotalia guianensis* | Coastal | Caraguatatuba/SP | SAR | F | A | 3 |
| MM098 | - |  | N/A | Autolysis | *Sotalia guianensis* | Coastal | Ubatuba/SP | SAR | M | A | 3 |
| MM099 | - |  | A | Slightly and diffusely engorged vessels | *Sotalia guianensis* | Coastal | Ubatuba/SP | SAR | M | A | 2 |
| MM100 | - |  | N/A | Autolysis | *Pontoporia blainvillei* | Coastal | Ubatuba/SP | SAR | M | F | 3 |
| MM101 | - |  | A | Slightly and diffusely engorged vessels | *Pontoporia blainvillei* | Coastal | Ubatuba/SP | SAR | F | F | 2 |
| MM102 | - |  | N/A | Autolysis | *Balaenoptera acutorostrata* | Oceanic | Ubatuba/SP | SAR | M | F | 3 |
| MM103 | + |  | N/A | Autolysis | *Pontoporia blainvillei* | Coastal | Ubatuba/SP | SAR | F | F | 2 |
| MM104 | - |  | A | Randomly pyknotic cortical tubules | *Kogia breviceps* | Oceanic | Ubatuba/SP | SAR | F | A | 2 |
| MM105 | - |  | A | Vessels moderately and diffusely engorged. Multifocal pyknotic cortical tubules. | *Sotalia guianensis* | Coastal | Ubatuba/SP | SAR | F | J | 2 |
| MM106 | - |  | A | Moderately and diffusely engorged vessels | *Sotalia guianensis* | Coastal | Ubatuba/SP | SAR | M | J | 2 |
| MM107 | - |  | A | Health tissue | *Sotalia guianensis* | Coastal | Ubatuba/SP | SAR | F | A | 3 |
| MM108 | - |  | A | Moderately and diffusely engorged vessels | *Pontoporia blainvillei* | Coastal | Ubatuba/SP | SAR | F | F | 2 |
| MM109 | - |  | N/A | N/A | *Pontoporia blainvillei* | Coastal | Ubatuba/SP | SAR | M | F | 3 |
| MM110 | + | LSQ | N/A | N/A | *Sotalia guianensis* | Coastal | Ubatuba/SP | SAR | M | J | 3 |
| MM111 | - |  | N/A | Autolysis | *Pontoporia blainvillei* | Coastal | Caraguatatuba/SP | SAR | M | F | 3 |
| MM112 | - |  | A | Vessels markedly and diffusely engorged. | *Sotalia guianensis* | Coastal | Caraguatatuba/SP | SAR | M | J | 2 |
| MM113 | - |  | A | Mild multifocal hyperemia. | *Pontoporia blainvillei* | Coastal | Caraguatatuba/SP | SAR | F | F | 3 |
| MM114 | - |  | A | Vessels moderately and diffusely engorged. | *Sotalia guianensis* | Coastal | Ubatuba/SP | SAR | F | J | 2 |
| MM115 | + | LSQ | A | Moderately and diffusely engorged vessels. | *Pontoporia blainvillei* | Coastal | Ubatuba/SP | SAR | F | J | 3 |
| MM120 | + | LSQ | B | Presence of multifocal areas of moderate hemorrhage and congestion. | *Stenella clymene* | Oceanic | Jandaraí/SP | SAR | F | J | 2 |
| MM121 | + | LSQ | B | Presence of multifocal areas of moderate hemorrhage and congestion. | *Sotalia guianensis* | Coastal | Aracaju/SE | WAR | M | A | 2 |
| MM122 | + | LSQ | A | Health tissue | *Sotalia guianensis* | Coastal | Estância/SE | WAR | F | A | 3 |
| MM123 | + | LSQ | B | Presence of multifocal areas of moderate hemorrhage and congestion. | *Sotalia guianensis* | Coastal | Pirambu/SE | WAR | F | A | 2 |
| MM124 | + | LSQ | A | Health tissue | *Sotalia guianensis* | Coastal | Aracaju/SE | WAR | M | F | 2 |
| MM125 | + | LSQ | N/A | N/A | *Sotalia guianensis* | Coastal | Aracaju/SE | WAR | F | A | 3 |

| **ID** | ***Lip*L32**  **PCR** | ***sec*Y sequencing** | **Status** | **Morphological Kidney description** | **Species** | **Habitat** | **City/State** | **Eco-Region** | **Sex** | **Age** | **COD** |
| --- | --- | --- | --- | --- | --- | --- | --- | --- | --- | --- | --- |
| MM126 | + | LSQ | N/A | N/A | *Sotalia guianensis* | Coastal | Barra dos Coqueiros/SE | WAR | F | J | 3 |
| MM127 | - |  | A | Health tissue | *Sotalia guianensis* | Coastal | Estância/SE | WAR | M | J | 2 |
| MM128 | + | *L. interrogans* | A | Health tissue | *Sotalia guianensis* | Coastal | Estância/SE | WAR | F | F | 2 |
| MM129 | - |  | N/A | N/A | *Sotalia guianensis* | Coastal | Aracaju/SE | WAR | M | A | 3 |
| MM130 | - |  | A | Health tissue | *Feresa attenuata* | Oceanic | Jandaraí/SE | WAR | F | J | 3 |
| MM131 | - |  | N/A | N/A | *Peponocephala electra* | Oceanic | Aracaju/SE | WAR | F | A | 2 |
| MM132 | + | LSQ | N/A | N/A | *Sotalia guianensis* | Coastal | Barra dos Coqueiros/SE | WAR | M | J | 3 |
| MM133 | - |  | A | Health tissue | *Sotalia guianensis* | Coastal | Barra dos Coqueiros/SE | WAR | F | J | 2 |
| MM134 | - |  | N/A | N/A | *Sotalia guianensis* | Coastal | Jandaraí/SE | WAR | M | A | 3 |
| MM135 | - |  | A | Health tissue | *Feresa attenuata* | Oceanic | Jandaraí/SE | WAR | M | J | 2 |
| MM136 | + | LSQ | A | Health tissue | *Sotalia guianensis* | Coastal | Jandaraí/SE | WAR | F | J | 2 |
| MM137 | - |  | N/A | N/A | *Peponocephala electra* | Oceanic | Aracaju/SE | WAR | M | F | 3 |
| MM138 | - |  | A | Health tissue | *Kogia sima* | Oceanic | Jandaraí/SE | WAR | F | A | 3 |
| MM139 | - |  | A | Health tissue | *Sotalia guianensis* | Coastal | Aracaju/SE | WAR | F | J | 2 |
| MM140 | - |  | A | Discrete, multifocal renal tubular degeneration. | *Sotalia guianensis* | Coastal | Barra dos Coqueiros/SE | WAR | M | F | 2 |
| MM141 | - |  | A | Health tissue | *Sotalia guianensis* | Coastal | Aracaju/SE | WAR | M | J | 2 |
| MM143 | - |  | S | Multifocal necrotic lymphocytic interial necromia and necrotic areas of tubular hemorrhage. | *Megaptera novaeangliae* | Oceanic | Macéio/AL | WAR | M | N | 2 |
| MM144 | - |  | N/A | N/A | *Kogia sima* | Oceanic | Feliz Deserto/AL | WAR | M | A | 2 |
| MM145 | - |  | N/A | N/A | *Sotalia guianensis* | Coastal | Passo de Camaragibe/AL | WAR | F | F | 2 |
| MM146 | - |  | N/A | N/A | *Megaptera novaeangliae* | Oceanic | Roteiro/AL | WAR | M | N | 2 |
| MM147 | - |  | A | Focal renal hemorrhage and formation of tubular casts | *Peponocephala electra* | Oceanic | Roteiro/AL | WAR | F | A | 2 |
| MM148 | - |  | N/A | N/A | *Balaenoptera physalus* | Oceanic | Barra de Santo Antônio/AL | WAR | F | J | 3 |
| MM149 | - |  | N/A | N/A | *Sotalia guianensis* | Coastal | Macéio/AL | WAR | F | A | 3 |
| MM150 | - |  | N/A | N/A | *Sotalia guianensis* | Coastal | Macéio/AL | WAR | M | J | 2 |
| MM151 | - |  | A | N/A | *Sotalia guianensis* | Coastal | Feliz Deserto/AL | WAR | M | A | 2 |

| **ID** | ***Lip*L32**  **PCR** | ***sec*Y sequencing** | **Status** | **Morphological Kidney description** | **Species** | **Habitat** | **City/State** | **Eco-Region** | **Sex** | **Age** | **COD** |
| --- | --- | --- | --- | --- | --- | --- | --- | --- | --- | --- | --- |
| MM152 | - |  | N/A | N/A | *Megaptera novaeangliae* | Oceanic | Japaratinga/AL | WAR | M | J | 3 |
| MM153 | - |  | N/A | N/A | *Sotalia guianensis* | Coastal | Roteiro/AL | WAR | M | F | 4 |
| MM154 | - |  | N/A | N/A | *Sotalia guianensis* | Coastal | Macéio/AL | WAR | F | A | 2 |
| MM155 | - |  | N/A | N/A | *Sotalia guianensis* | Coastal | Feliz Deserto/AL | WAR | M | J | 3 |
| MM156 | - |  | B | Moderate multifocal necrohemorrhagic nephritis | *Sotalia guianensis* | Coastal | Macéio/AL | WAR | M | J | 2 |
| MM157 | - |  | N/A | N/A | *Sotalia guianensis* | Coastal | Jequiá da Praia/AL | WAR | M | A | 3 |
| MM158 | - |  | N/A | N/A | *Sotalia guianensis* | Coastal | Roteiro/AL | WAR | M | J | 2 |
| MM159 | - |  | N/A | N/A | *Sotalia guianensis* | Coastal | Marechal Deodoro/AL | WAR | F | J | 3 |
| MM160 | - |  | N/A | N/A | *Sotalia guianensis* | Coastal | Coruripe/AL | WAR | F | A | 3 |
| MM161 | - |  | N/A | N/A | *Peponocephala electra* | Oceanic | São Miguel do Milagres/AL | WAR | F | J | 3 |
| MM162 | - |  | N/A | N/A | *Sotalia guianensis* | Coastal | Roteiro/AL | WAR | M | F | 3 |
| MM163 | - |  | N/A | N/A | *Megaptera novaeangliae* | Oceanic | Feliz Deserto/AL | WAR | M | J | 2 |
| MM164 | - |  | A | Health tissue | *Sotalia guianensis* | Coastal | Coruripe/AL | WAR | M | J | 3 |
| MM166 | - |  | N/A | N/A | *Tursiops truncatus* | Oceanic | Icapuí/CE | WAR | F | A | 3 |
| MM167 | - |  | N/A | N/A | *Sotalia guianensis* | Coastal | Fortaleza/CE | WAR | F | A | 2 |
| MM168 | - |  | N/A | Areas of bleeding / Autolysis | *Kogia breviceps* | Oceanic | Fortaleza/CE | WAR | M | A | 3 |
| MM169 | - |  | N/A | Autolysis | *Stenella longirostris* | Oceanic | Camocim/CE | WAR | F | A | 2 |
| MM170 | - |  | A | Multifocal hemorrhage, Multifocal interstitial nephritis ; Diffuse congestion with acute tubular degeneration dispersion. | *Sotalia guianensis* | Coastal | Icapui/CE | WAR | M | J | 2 |
| MM171 | - |  | N/A | N/A | *Feresa attenuata* | Oceanic | Trairí/CE | WAR | F | F | 3 |
| MM172 | - |  | B | Extensive necrosis and hematic pigmentation of the renal tubules. | *Sotalia guianensis* | Coastal | Aracati/CE | WAR | F | A | 2 |
| MM173 | - |  | N/A | Areas of bleeding / Autolysis | *Stenella attenuata* | Oceanic | Caucaia/CE | WAR | F | A | 3 |
| MM174 | - |  | B | Mild hydropic degeneration of the renal tubular epithelium. Irregular foci of calcification in the pelvis. Moderate congestion. | *Globicephala macrorhynchus* | Oceanic | Trairí/CE | WAR | F | A | 3 |
| MM175 | - |  | B | multifocal moderate acute tubular degeneration; congestion; tubular proteinosis and diffuse hemorrhage | *Stenella coeruleoalba* | Oceanic | Caucaia/CE | WAR | F | N | 2 |
| MM176 | - |  | A | Congestion | *Grampus griseus* | Oceanic | Icapuí/CE | WAR | F | A | 2 |
| MM177 | - |  | N/A | N/A | *Megaptera novaeangliae* | Oceanic | Trairí/CE | WAR | F | A | 2 |

| **ID** | ***Lip*L32**  **PCR** | ***sec*Y sequencing** | **Status** | **Morphological Kidney description** | **Species** | **Habitat** | **City/State** | **Eco-Region** | **Sex** | **Age** | **COD** |
| --- | --- | --- | --- | --- | --- | --- | --- | --- | --- | --- | --- |
| MM178 | - |  | A | Health tissue | *Feresa attenuata* | Oceanic | São Gonçalo do Amarante/CE | WAR | M | A | 2 |
| MM179 | - |  | N/A | N/A | *Stenella longirostris* | Oceanic | Acaraú/CE | WAR | F | A | 3 |
| MM180 | - |  | N/A | N/A | *Sotalia guianensis* | Coastal | Paraipaba/CE | WAR | M | F | 2 |
| MM181 | - |  | A | Mild hydropic degeneration. Congestion | *Kogia sima* | Oceanic | Beberibe/CE | WAR | M | A | 2 |
| MM182 | - |  | A | Congestion | *Kogia sima* | Oceanic | Itarema/CE | WAR | M | A | 3 |
| MM183 | - |  | B | Moderate hydropic degeneration of the renal tubular epithelium. Congestion | *Feresa attenuata* | Oceanic | Cruz/CE | WAR | F | F | 2 |
| MM184 | - |  | N/A | N/A | *Globicephala macrorhynchus* | Oceanic | Icapuí/CE | WAR | F | N | 2 |
| MM185 | - |  | A | Mild hydropic degeneration of the tubular epithelium. Congestion | *Sotalia guianensis* | Coastal | São Gonçalo do Amarante/CE | WAR | F | A | 2 |
| MM186 | - |  | N/A | N/A | *Stenella coeruleoalba* | Oceanic | Caucaia/CE | WAR | F | J | 2 |
| MM187 | - |  | N/A | N/A | *Kogia breviceps* | Oceanic | Amontada/CE | WAR | M | A | 3 |
| MM188 | - |  | A | Subcapsular hemorrhage. Congestion | *Kogia sima* | Oceanic | Icapuí/CE | WAR | F | A | 2 |
| MM189 | - |  | N/A | N/A | *Sotalia guianensis* | Coastal | Caucaia/CE | WAR | M | F | 3 |
| MM190 | - |  | N/A | N/A | *Sotalia guianensis* | Coastal | Fortaleza/CE | WAR | M | F | 2 |
| MM191 | - |  | N/A | Autolysis areas. Congestion | *Globicephala macrorhynchus* | Oceanic | Beberibe/CE | WAR | F | J | 2 |
| MM192 | - |  | N/A | N/A | *Globicephala macrorhynchus* | Oceanic | Icapuí/CE | WAR | F | F | 2 |
| MM192 | - |  | N/A | N/A | *Stenella clymene* | Oceanic | Aquiraz/CE | WAR | M | A | 2 |
